# Prevalence and Molecular Genetics of Methicillin-Resistant *Staphylococcus aureus* Colonization in Nursing Homes in Saudi Arabia

**DOI:** 10.1155/2020/2434350

**Published:** 2020-06-03

**Authors:** Ahmed Albarrag, Ashwag Shami, Abrar Almutairi, Sara Alsudairi, Sumayh Aldakeel, Amani Al-Amodi

**Affiliations:** ^1^Department of Pathology, School of Medicine, King Saud University, Riyadh, Saudi Arabia; ^2^College of Science, Princess Nourah Bint Abdulrahman University, Riyadh, Saudi Arabia

## Abstract

**Objective:**

Methicillin-resistant *Staphylococcus aureus* (MRSA) is one of the main causative agents of nosocomial infections that has posed a major threat to those with compromised immune systems such as nursing home residents. The aim of this study was to determine the rates of MRSA strains and the types of *Staphylococcal Cassette Chromosome mec (SCCmec*)in nursing homes in Saudi Arabia.

**Methods:**

A total of 188 nasal swabs were collected from the residents and nursing staff in two nursing homes in Riyadh, Saudi Arabia. All MRSA isolates were tested for antimicrobial susceptibility and analyzed for *mecA* and *SCCmec* typing by multiplex PCR assay. Detection of the Panton–Valentine leukocidin (*PVL*) gene was also tested in all positive MRSA isolates by multiplex PCR using specific primers.

**Results:**

Among the 188 collected nasal swabs (105 males and 83 females), MRSA colonization rate was 9.04% (11 (5.85%) females and 6 (5.71%) males). About 47% of MRSA were multidrug resistant (MDR) as acquired resistance to beta-lactam, macrolide, and aminoglycoside antibiotics. However, all the MRSA isolates showed susceptibility to vancomycin, tigecycline, and linezolid. All the MRSA isolates (*n* = 17) were *mecA*-positive with the SCC*mec* IVc (*n* = 7, 41.18%) as the most common SCC*mec* type followed by SCC*mec* V (*n* = 5, 29.41%) and SCC*mec* IVa (*n* = 2, 11.76%). The remaining isolates (*n* = 3) were nontypeable (17.65%). In addition, the PVL toxin gene was only detected in four of the male samples.

**Conclusion:**

MRSA nasal colonization is a common incident among nursing home residents. The prevalence of community-associated (CA) MRSA (SCC*mec* IV and V) was more common than hospital-associated (HA) MRSA in our study samples. It is crucial to investigate such rate of incidence, which is a key tool in preventive medicine and would aid in determining health policy and predict emergent outbreaks.

## 1. Introduction

*Staphylococcus aureus*, especially methicillin-resistant *S. aureus* (MRSA), is one of the main nosocomial pathogens associated with morbidity and mortality in both hospital and community settings [1]. According to the Center for Disease Control and Prevention (CDC), MRSA have resulted in almost 11,000 deaths in 2011 [2]. The prevalence of MRSA in nursing homes varied substantially from 1% to 23% between countries in Europe [3]. Nursing homes have been denoted to increase MRSA transmission and infection due to their propensity to admit old-age residents with premorbid conditions and weakened immune systems [4–6]. A 2005 cross-sectional survey carried out by a Belgian cohort demonstrated that, on average, 19% of the screened nursing home residents were MRSA carriers [7].

*S. aureus* isolates have gained resistance to methicillin due to the Staphylococcal cassette chromosome *SCCmec* genetic element integrated into *S. aureus* genomic chromosomal DNA downstream the *orfX* gene [8]. This cassette contains the methicillin resistance (*mecA*) gene, which is responsible for resistance to antibiotics such as methicillin, penicillin, and other penicillin-like antibiotics and additional antibiotic resistance determinants [8]. Compellingly, studies have proven a strong correlation between MRSA strains and their accrued resistance to commonly used groups of antibiotics such as tetracycline, fluoroquinolones, aminoglycosides, chloramphenicol, and macrolides [9].

The level of resistance to non-*β*-lactam antibiotic classes varies between strains that are produced by either health care-associated (HA) MRSA or community-associated (CA) MRSA [10]. CA-MRSA strains differ from HA-MRSA genotypically and phenotypically. CA-MRSA harbors relatively smaller staphylococcal cassette chromosomal *mec* (*SCCmec*), which is a cassette that contains the *mecA* gene, type IV (21 to 24 kb) or type V (28 kb) [10–12]. In contrast, HA-MRSA strains usually contain larger *SCC mec* belonging to type I (34 kb), II (53 kb), or III (67 kb) [12]. These larger elements also contain the *mecA* gene and are resistant to more non-*β*-lactam classes of antimicrobials [10]. PVL is a virulence factor, which belonging to the family of synergohymenotropic toxins, which has an important role in pathogenicity by producing pores in the membrane of host defense cells [12].

The aim of this study is to investigate the rate of MRSA colonization in nursing homes in Saudi Arabia by isolating *S. aureus* and molecular identification of the of *SCCmec* types, detection of the *mecA* resistance gene, and the virulence gene *PVL*.

## 2. Materials and Methods

### 2.1. Sample Collection

This study was authorized by the Medical Ethic Committee of Princess Nourah Bin Abdulrahman University (IRB number 17-0046). A total of 188 individuals (105 males and 83 females) aged from 39 up to 104 residing in a nursing home in Riyadh, Saudi Arabia, were screened for MRSA colonization after obtaining informed consent from the residents and nursing staff. Amies transport swabs were used to sample the anterior nares of residents. The swabs were processed within 2 hours of collection. All swabs were cultured/cultivated on mannitol salt agar with oxacillin (MSAO) media for 24 hrs at 37°C to screen for MRSA. Following 24 hours of incubation, presumptive MRSA colonies were subcultured on blood agar for further identification by coagulase testing and standard microbiological methods.

### 2.2. Identification and Antimicrobial Susceptibility Testing

Identification and antimicrobial susceptibility testing was performed on all presumptive MRSA isolates by the automated method VITEK 2 (BioMérieux, Inc., Durham, NC) using AST580-GP in accordance with the manufacturer's instructions. The interpretation of susceptibility testing was performed based on the clinical breakpoints following Clinical and Laboratory Standards Institute (CLSI).

### 2.3. *SCCmec* Typing and PCR-Based Assays for the *PVL* Gene

Genomic DNA extraction from the MRSA isolates was done using QIAamp DNA Mini Kit (Qiagen, Germany) in accordance with manufacturers' instructions. The template DNA was used in a multiplex polymerase chain reaction (mPCR) to characterize and identify the Staphylococcal Cassette Chromosome (*SCC*) *mec* types I–V for MRSA as described by McClure-Warnier [13]. Additionally, a multiplex PCR assay was performed that targets the 16S rRNA gene, the *lukS/F-PV* genes, which encode PVL, and the *mecA* gene as described by McClure et al. [14]. All PCR products were visualized on a 1% agarose gel stained with ethidium bromide.

## 3. Results

Among the 188 collected nasal swabs, 20 swabs grown presumptive MRSA colonies on mannitol salt agar with oxacillin (MSAO). Of these 20 samples, 17 were coagulase-positive, which further identified as *S. aureus* using the VITEK 2 automated identification system. This indicates a 9.04% rate of nasal colonization with MRSA. Among the 17 MRSA carriers, 11 (13.25% (11/83)) were females and 6 (5.71% (6/105)) were males with a mean age of 62.45 (age range 39–104 years).

The antimicrobial susceptibility testing by the VITEK 2 automated method is determined for benzylpenicillin, cloxacillin, oxacillin, cefaclor, erythromycin, clindamycin, gentamicin, fosfomycin, levofloxacin, teicoplanin, tetracycline, vancomycin, tigecycline, and linezolid (Table 1). About 47% of MRSA were multidrug resistant (MDR) as acquired resistance to beta-lactam, macrolide, and aminoglycoside antibiotics. However, all the MRSA isolates showed susceptibility to vancomycin, tigecycline, and linezolid.

**Figure 1:**
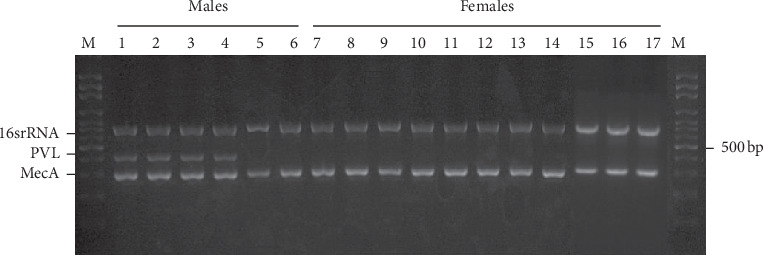
Multiplex PCR assay for the detection of PVL and *mecA* genes. M: 100 bp DNA marker, lanes 1 to 6: MRSA isolates from men, and lanes 7 to 17: MRSA isolates from women. 16 srRNA: 16S rRNA gene amplicon (762 bp), PVL: *lukS/F-PV* amplicon (433 bp), and MecA: *mecA* amplicon (310 bp).

Identification of methicillin resistance strains was confirmed by the detection of the *mecA* gene in a multiplex PCR. All the 17 MRSA isolates had a PCR product of 310 bp corresponding to the size of the amplified DNA fragment of the *mecA* gene (Figure 1). SCC*mec* typing of these 17 MRSA isolates was performed by the multiplex PCR. The most common type among all isolates was SCC*mec* IVc (*n* = 7, 41.18%) followed by SCC*mec* V (*n* = 5, 29.41%) and SCC*mec* IVa (*n* = 2, 11.65%). The remaining isolates (*n* = 3) were nontypeable (17.65%) (Table 2).

**Figure 2:**
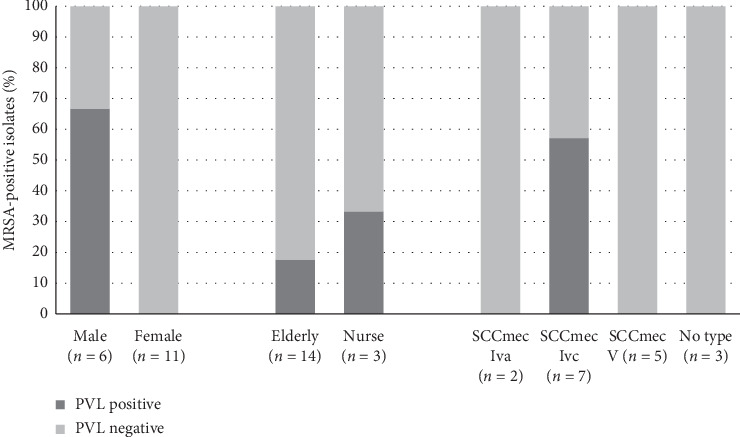
Frequency of methicillin-resistant *Staphylococcus aureus* (MRSA) colonization in positive and negative PVL isolates. Four male samples were positive for PVL (66.67% (4/6)). Three (21.43% (3/14)) were elderly and one (33.33% (1/3)) was a nurse. The 4 PVL-positive isolates were all SCC*mec*-IVc (57.14% (4/7)).

In addition, the virulence gene, PVL, was also detected (Figure 1). Of the 17 MRSA isolates, four of the male samples (66.67% (4/6)) were PVL-positive, while all the female samples were PVL-negative. Three of the PVL-positive isolates were elderly individuals and one was a nurse, yet all the four isolates had the SCC*mec* IVc subtype (57.14% (4/7)) (Figure 2).

## 4. Discussion

Controlling MRSA strains' dissemination within nursing homes is challenging [15]. The documented prevalence rates of MRSA in nursing homes vary from 1–23% [3, 16]. In our study, the prevalence rate of MRSA colonization in nursing homes is 9%, which characterizes a moderate threat. Effective approaches such as surveillance assessments of MRSA classification are required to meet the need of controlling MRSA dissemination. This could be achieved through the detection of *mecA* gene carriers and all the SCC*mec* types. This is the first study to be conducted in Saudi Arabia aiming to assess nasal carriage prevalence of MRSA colonization in residential care homes for the elderly through molecular identification of *SCCmec* typing, *mecA* resistance gene, and virulence gene PVL.

Our data demonstrated that gender was not associated with MRSA colonization compared to previous studies, where males were predominantly associated with MRSA colonization [17–20]. The resistance profile revealed lower resistance rates compared to the average of resistance reported previously especially for gentamicin, erythromycin, tetracycline, fosfomycin, and linezolid [9].

Since all the isolates were oxacillin-resistant, it was expected to observe the acquisition of the methicillin resistance gene *mecA* in all isolates. SCC*mec* typing was performed on the isolates in which 10 (66.6%) were SCC*mec* type IV and 5 (33.3%) were SCC*mec* type V. These two most common types of SCC*mec*, IV and V, were carried by MRSA strains presumably due to their relatively small size that facilitates their spread among MRSA strains [21]. Reported MRSA SCC*mec* types have no influence on the antibiotic resistant pattern of MRSA isolates. In addition to the *mec*A gene, four isolates were carrying the PVL gene which is linked with virulence; the PVL toxin has been associated with many of the severe clinical presentations of MRSA infection.

It is worth noting that based on the molecular analysis of SCC*mec*, isolates show hallmarks of being a community-associated (CA) MRSA strain type. The frequent admissions of institutionalized elderly members of the community to the acute care hospitals expose them regularly to MRSA dissemination. However, in our study, none of the MRSA isolates were harboring health care-associated (HA) MRSA strain types; in contrast, subjects were all colonized by either *SCCmec* IV or V types. Since these cassettes are mainly associated with (CA) MRSA, it is hard to rule out the emergence of MRSA into this long-term care facility from the community. This has been documented worldwide by others who have reported a slow emergence of CA-MRSA strain types in nursing homes [6, 22–25]. On the contrary, PVL toxin gene is a community marker, and it was observed in 23.53% (4/17) of the isolates, which is relatively low compared to the rates of carriage of the PVL in CA-MRSA strains, >75% [26]. Collectively, this would all suggest an acquisition of a concurrent distribution of both community- and hospital-associated MRSA in nursing homes. Further studies using well-established methods such as pulse-field gel electrophoresis (PFGE) or staphylococcal protein A (spa) typing methods to genotype MRSA isolates would facilitate determining strain relatedness, evolutionary patterns, and source of transmission more accurately [27].

The limitations of our study were mainly driven by including a relative small number of subjects especially with respect to MRSA colonization. Larger sample sizes from multiple community nursing homes are required to accurately depict the picture of MRSA prevalence in Saudi Arabia. Additionally, the extent of CA-MRSA colonization among nursing home residents cannot be determined as this study was not conducted as a point prevalence survey that includes all the nursing home residents. However, the revealed data raise the concern regarding the introduction of these strain types to the long-term care residents.

This study showed incidence of nasal carriage of CA-MRSA in nursing homes in Saudi Arabia denoting an essential indicator of strain prevalence. Investigation of such rate of incidence is a key tool in preventive medicine, assisting health care legislators in determining health policy and predicting emergent outbreaks. The introduction of CA-MRSA strains in this setting will likely change the selection of empirical antibiotic therapies for MRSA infections. Also, it could pose growing problems in the severity of these staphylococcal infections in the immunocompromised residents.

## Figures and Tables

**Table 1:** Antibiotic resistant profile of MRSA isolates from elderly residents and nurses.

Antimicrobial agent	MIC range (*μ*g/mL)	Resistant (*n* = 17) *n* (%)	MRSA SCC*mec* types (*n* = 14)		
			IVa (*n* = 2) *n* (% R)	IVc (*n* = 7) *n* (% R)	V (*n* = 5) *n* (% R)
Benzylpenicillin	>= 0.5	17 (100)	2 (100)	7 (100)	5 (100)
Cloxacillin	>= 0.5	17 (100)	2 (100)	7 (100)	5 (100)
Oxacillin	0.25–4	17 (100)	2 (100)	7 (100)	5 (100)
Cefaclor	0.25–4	17 (100)	2 (100)	7 (100)	5 (100)
Gentamicin	0.50–16	8 (47.06)	1 (50)	4 (57.14)	1 (20)
Levofloxacin	0.12–8	3 (17.65)	0 (0.0)	1 (14.28)	1 (20)
Erythromycin	0.25–8	10 (58.82)	2 (100)	5 (71.43)	2 (40)
Clindamycin	0.25–8	9 (52.94)	1 (50)	5 (71.43)	2 (40)
Teicoplanin	0.50–32	1 (5.88)	0 (0.0)	0 (0.0)	0 (0.0)
Vancomycin	0.50–2	0 (0.0)	0 (0.0)	0 (0.0)	0 (0.0)
Tetracycline	1–16	1 (5.88)	1 (50)	0 (0.0)	0 (0.0)
Tigecycline	0.12–0.5	0 (0.0)	0 (0.0)	0 (0.0)	0 (0.0)
Fosfomycin	8–128	4 (23.52)	1 (50)	2 (28.57)	0 (0.0)
Linezolid	1–4	0 (0.0)	0 (0.0)	0 (0.0)	0 (0.0)

**Table 2:** *mecA* gene, PVL gene, and *SCCmec* types in MRSA isolates.

Sample ID	Gender	Residential type	*SCCmec* type	*mecA* gene	PVL gene
1	Male	E	IVc	+	+
2		E	IVc	+	+
3		E	IVc	+	+
4		N	IVc	+	+
5		N	—	+	−
6		N	IVc	+	−

7	Female	E	V	+	−
8		E	V	+	−
9		E	IVa	+	−
10		E	V	+	−
11		E	V	+	−
12		E	IVa	+	−
13		E	V	+	−
14		E	—	+	−
15		E	IVc	+	−
16		E	IVc	+	−
17		E	—	+	−

E: elderly and N: nurse.

## Data Availability

All data that were used to support the findings of this study are included within the article.
